# Patch-Type Vibration Visualization (PVV) Sensor System Based on Triboelectric Effect

**DOI:** 10.3390/s21123976

**Published:** 2021-06-09

**Authors:** Sun-Jin Kim, Myeong-Lok Seol, Byun-Young Chung, Dae-Sic Jang, Jong-Hwan Kim, Young-Chul Choi

**Affiliations:** 1 Smart Structural Safety and Prognosis Research Division, Korea Atomic Energy Research Institute, 111 Daedeok-daero 989Beon-gil, Yuseong-gu, Daejeon 34057, Korea; cby@kaeri.re.kr (B.-Y.C.); bigplant@kaeri.re.kr (D.-S.J.); jhkim0630@kaeri.re.kr (J.-H.K.); cyc@kaeri.re.kr (Y.-C.C.); 2 Center for Nanotechnology and Universities Space Research Association, NASA Ames Research Center, Moffett Field, CA 94035, USA; myeonglok.seol@nasa.gov

**Keywords:** self-sustainable vibration visualization sensor system, triboelectric effect, condition monitoring, signal-to-pattern encoding, pattern-to-data decoding

## Abstract

Self-powered wireless sensor systems have emerged as an important topic for condition monitoring in nuclear power plants. However, commercial wireless sensor systems still cannot be fully self-sustainable due to the high power consumption caused by excessive signal processing in a mini-electronic computing system. In this sense, it is essential not only to integrate the sensor system with energy-harvesting devices but also to develop simple data processing methods for low power schemes. In this paper, we report a patch-type vibration visualization (PVV) sensor system based on the triboelectric effect and a visualization technique for self-sustainable operation. The PVV sensor system composed of a polyethylene terephthalate (PET)/Al/LCD screen directly converts the triboelectric signal into an informative black pattern on the LCD screen without excessive signal processing, enabling extremely low power operation. In addition, a proposed image processing method reconverts the black patterns to frequency and acceleration values through a remote-control camera. With these simple signal-to-pattern conversion and pattern-to-data reconversion techniques, a vibration visualization sensor network has successfully been demonstrated.

## 1. Introduction

Condition monitoring systems in nuclear power plants are critical for anomaly detection and management of operational transients and accidents [1,2,3]. Among them, vibration monitoring for primary coolant and secondary loop systems including the reactor, steam generator, pressurizer, heat exchanger, turbine, and generator is critical. Enhanced vibration monitoring systems such as an acoustic leak monitoring system (ALMS), reactor coolant pump vibration monitoring system (RCPVMS), internal vibration monitoring system (IVMS), and loose parts monitoring system (LPMS) thus far have been developed using acceleration, acoustic emission (AE), and displacement sensors during the past few decades [4,5,6,7,8,9]. In addition, many fault diagnoses of machinery using high vibration analysis have been proposed [10,11,12]. These systems provide data processing and visualization to a nuclear power plant control room to assist in making correct decisions and rapid responses on the detected issues. However, it is challenging to electrically wire and maintain hundreds of vibration sensors in the hard-to-access and dangerous locations of a plant. In this sense, self-powered and self-sustaining sensor units without needs of electrical wiring, battery changing, and additional maintenance are actively being investigated for the next generation wireless sensor systems. Recently, wireless sensor systems integrated with energy-harvesting devices such as solar cells [13,14], piezoelectric micro-generators [15,16], and thermoelectric generators [17,18] have been reported for realizing self-powered sensor systems, but it remains challenging to meet the realistic requirements. This is because commercial wireless sensor systems still consume high electricity due to excessive signal processing steps: vibration signal detection, analog-to-digital conversion, fast Fourier transform (FFT), and data transmission to a control room. In addition, the use of wireless sensor systems has been restricted by safety regulations in nuclear power plants due to electromagnetic interference (EMI) that can cause electrical systems to malfunction. For these reasons, it is essential not only to integrate the sensor system with energy-harvesting devices but also to develop simple data processing methods for low power schemes.

In this paper, we propose patch-type vibration visualization (PVV) sensor system based on the triboelectric effect and a visualization technique enabling genuine self-sustainable operation. The triboelectric effect is based on the phenomenon of static electricity (triboelectricity), which is created by physical contact between two surfaces. With triboelectric energy harvesting, input mechanical energy sources such as motion, wind, wave, sound, and vibration activate the static electricity to generate an output electrical signal [19,20,21,22,23,24]. Static electricity is generated on its own only by physical contact, and thus no additional energy supply is required. In addition, triboelectric sensor probes offer a wide material choice window, light weight, high robustness, low cost, and high structural variability [25,26]. For vibration sensing on arbitrary surfaces in a nuclear power plant, the structure must be thin and flexible so that it can be applied to various geometries. Considering these requirements, a prototype composed of a polyethylene terephthalate (PET)/Al/LCD screen is fabricated as a PVV sensor system, and its frequency and acceleration dependencies are evaluated. It is demonstrated that the PVV sensor system converts triboelectric signals to a black pattern on the LCD screen without the need of excessive signal processing, enabling an extremely low power scheme. Subsequently, a pattern-to-data reconversion technique that quantitatively decodes the black patterns to frequency and amplitude values is demonstrated through remote camera monitoring and an image processing algorithm.

## 2. Materials and Methods

### 2.1. Materials and Operating Principle

**Figure 1:**
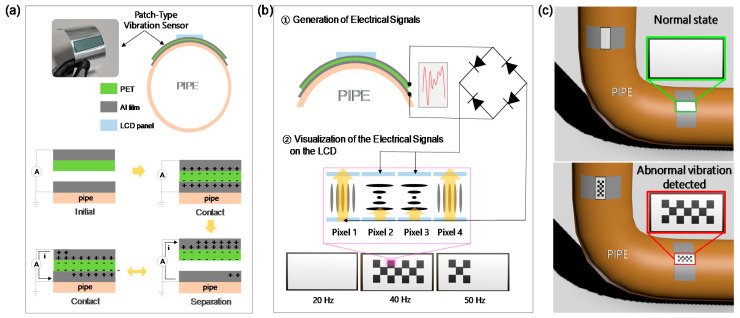
Schematic illustration and working mechanism of (**a**) the PVV sensor probe and (**b**) the visualization system. (**c**) Example of the PVV sensor system for detecting abnormal vibration signals on a coolant pipe.

The proposed PVV sensor with dimensions of 60 mm × 155 mm × 0.24 mm has a vertically stacked structure with separated top and bottom parts (Figure 1a). Details of fabrication process of the PVV sensor system are illustrated in Figure S1. The bottom layer consists of a flexible Al film placed on the target surface. The Al layer simultaneously serves as the triboelectric layer and the bottom electrode. The top part is composed of the PET triboelectric layer and the Al top electrode. The two parts are separated by a gap between them and the size of the gap changes when there is external vibration. The operation of the PVV sensor probe follows that of a contact-separation mode triboelectric nanogenerator (TENG) [27,28,29]. External vibration makes the top PET/Al layer vibrate vertically, causing repeated contact and separation with the Al bottom layer. The contact electrification is activated from the initial contact, and the PET thereby receives negative fixed charges and the Al receives positive mobile charges [30,31,32]. As the top layer separates from the Al bottom layer, the negative fixed charges at the PET attract their positive counter charges to the Al top electrode. In this case, the mobile positive charges at the Al bottom layer move to the top electrode, creating an output current. When the top layer approaches the bottom layer, the fixed charges at PET bring the positive counter charges to the bottom electrode, creating a current in the opposite direction. Therefore, the vertical vibration of the top part, which is the input mechanical energy, generates an alternating current between the two electrodes, which is the output electrical energy. Figure 1b presents the proposed vibration visualization approach combining the PVV sensor probe and the LCD screen. The LCD screen is fixed on the outer surface of the PVV sensor probe, and is electrically connected to the top and bottom electrodes. Triboelectric signals generated by the PVV sensor probe are transferred directly to the LCD screen and turn on the pixel array to show a black pattern. The vibration frequency and acceleration amplitude determine the amounts of triboelectric charges and current that are generated, and different types of patterns are thereby displayed on the LCD screen. This technique thus allows us to simply recognize changes in the frequency and acceleration amplitude of the target surface through the LCD screen. Figure 1c describes the use of the PVV sensor system on a coolant loop pipe in a nuclear power plant. The sensor displays white color in the normal state but shows the black pattern when abnormal signals are detected.

### 2.2. Characerization and Measurement

**Figure 2:**
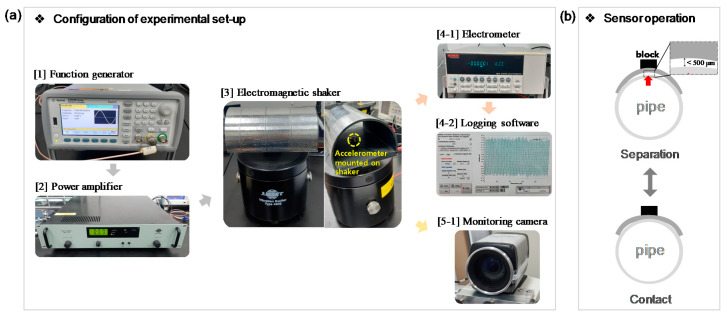
(**a**) Configuration of experimental setup. (**b**) Schematic illustration of the PVV sensor operation.

To evaluate the output characteristics of the PVV sensor system, we set up a vibration test bed with a vibration exciter (B&K type 4809) and a 3D printed cylinder pipe that mimics an actual coolant loop (Figure 2a). A function generator (Agilent 33500B series) and a power amplifier (B&K type 2718) were used to drive the test bed and to control vibration frequency and amplitude. In addition, a commercial sub-miniature piezoelectric charge accelerometer (B&K type 4375) was placed at the cylindrical substrate to refer actual frequency and acceleration amplitude of the test bed under excitation. Open-circuit voltage (VOC) and short-circuit current (ISC) generated by the PVV sensor probe were measured by an electrometer (Keithley 6514), which has extremely high input impedance on voltage measurements. All the measurements were conducted at temperature of 21.5 °C and humidity of 32.1%.

## 3. Results

### 3.1. Resonant Frequency Tuning

**Figure 3:**
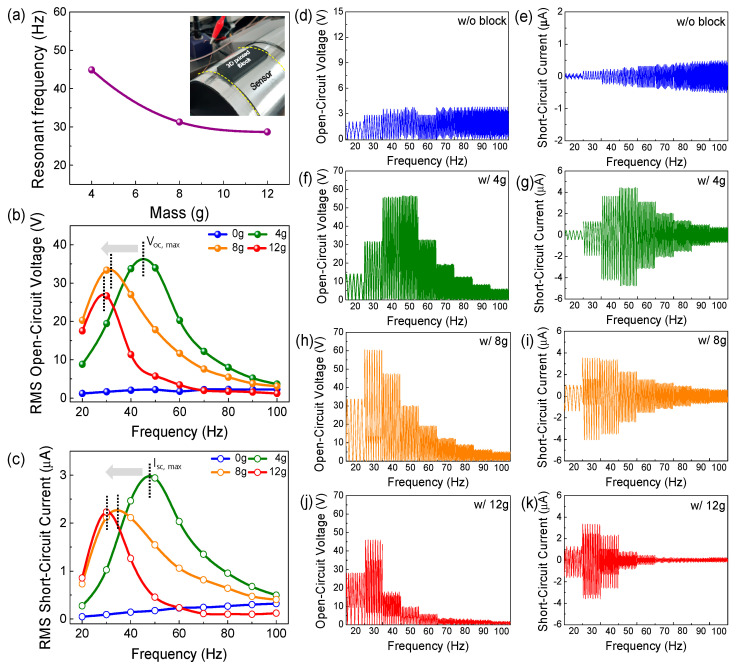
Output characteristics of the mass-loaded PVV sensor probe depending on the block mass: (**a**) Resonant frequency shift, (**b**) RMS open-circuit voltage (RMS V_oc_), (**c**) RMS short-circuit current (RMS I_sc_), and (**d**–**k**) Raw data of individual open-circuit voltages and currents when the block mass is (**d**,**e**) 0 g, (**f**,**g**) 4 g, (**h**,**i**) 8 g, and (**j**,**k**) 12 g.

In order to improve the sensitivity of the PVV sensor on a target surface, the resonant frequency of the sensor should be matched to the frequency range of the target surface, because the amplitude of electrical signals induced by the sensor probe steeply changes near the resonant frequency, as shown in Figure 3b. Here, we used a simple technique to match the resonant frequency of the PVV sensor to the frequency range of the target structure by mounting a 3D-printed mass on the PVV sensor probe (Figure 3a). The resonant frequency of the sensor can be adjusted with different masses corresponding to the natural frequency equation given below:

$$f_{n}=\frac{1}{2\pi }\cdot \sqrt{\frac{k}{m}}$$

where *f_n_* is the natural frequency of a simple mass-spring oscillator, *k* is the stiffness, and *m* is the mass. The natural frequency is proportional to the square root of the ratio of stiffness to mass and thus the resonant frequency of the PVV sensor can be simply modified. Although both stiffness and loading mass are controllable, changing the loading mass is simpler and more accurate in most practical scenarios, so we focused more on the mass dependence. Three different masses (4 g, 8 g, and 12 g) were mounted on the outer surface of the sensor probe and their frequency characteristics were measured. As shown in Figure 3b,c, the maximum peak voltage and current generated by the sensor probe are clearly changed depending on the mass. In particular, the sensor with the 4 g block shows a maximum resonant frequency of 45 Hz with a root-mean-square (RMS) V_oc_ of 36 V and RMS I_sc_ of 2.95 μA. The peaks gradually shift from 45 to 30 Hz with heavier mass, which follows the natural frequency equation. We add a graph of power densities at the resonant frequencies as a function of load resistance in Figure S2. However, the maximum voltage and current peaks and resonant frequency for the bare sensor probe are not found in the frequency range from 10 to 100 Hz owing to the extremely low weight of the sensor probe. With this approach, the resonant frequency of the PVV sensor probe can be simply adjusted depending on various vibration environments. In addition, multiple PVV sensors with different loading masses can be used to configure a multi-sensor system when we want to detect a wide frequency range. In this case, the loading masses have to be determined according to the vibration frequency range that we want to detect.

### 3.2. Image Signal Encoding/Decoding Technique

**Figure 4:**
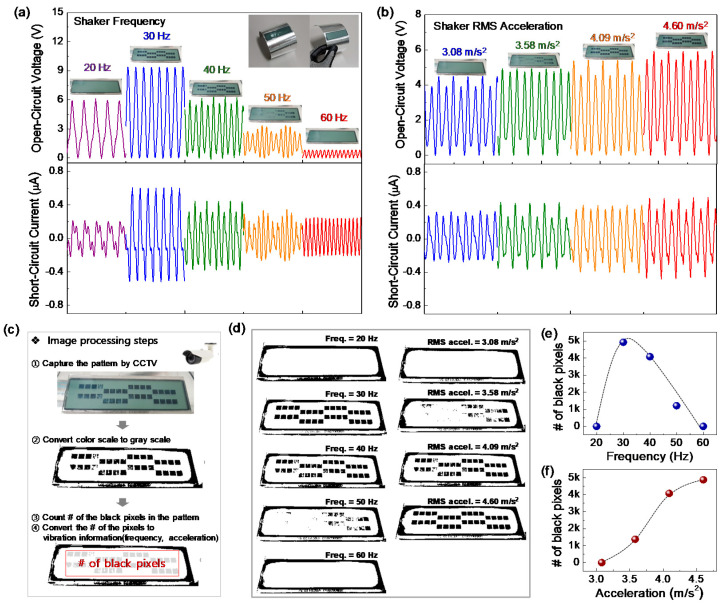
Signal-to-pattern conversion and pattern-to-data reconversion techniques. Signal-to-pattern conversion of a PVV sensor system under various (**a**) frequencies and (**b**) acceleration amplitudes. (**c**) Image processing steps for the pattern-to-data reconversion. (**d**) Color scale to gray scale conversion steps using OpenCV. (**e**,**f**) Relationship between the numbers of black pixels under various vibration frequencies and acceleration amplitudes.

To verify the performance of the PVV sensor system and its compatibility with vibration monitoring in industrial areas, signal-to-pattern conversion (vibration encoding to pattern) and pattern-to-data reconversion (pattern decoding to frequency and acceleration) techniques are developed under various frequencies and acceleration amplitudes. The black pattern on the LCD screen fully turns on at the resonant frequency of 30 Hz (Figure 4a) due to high triboelectric power generation. When the vibration frequency slightly shifts to lower or higher frequency, some area of the pattern disappears due to decreased triboelectric power generation, and all of the pattern completely disappears below 20 Hz or above 60 Hz. Furthermore, the PVV sensor is very sensitive to the acceleration amplitude, ranging from 3.08 to 4.60 m/s^2^ at 40 Hz (Figure 4b). The pattern fully turns on at 4.60 m/s^2^, and partially turns off as the amplitude decreases down to 3.08 m/s^2^. From the simple pattern changes on the LCD screen, operation engineers can immediately perceive the vibration frequency and amplitude shifts of the target structure when unexpected structural and mechanical faults occur. 

In order to quantitatively decode the patterns on the LCD screen, an image processing algorithm has been developed using OpenCV with python, as illustrated in Figure 4c. First, original images on the LCD screen are captured by a remote camera and the image patterns are then converted to gray scale using the image processing algorithm. The numbers of black pixels in the red color box pattern are then automatically counted, as shown in Figure 4c. Figure 4d shows all the gray-scale images converted by the image processing algorithm under various vibration frequencies and acceleration amplitudes. As we expected, there is a clear correlation between the number of black pixels and the vibration properties (frequency and acceleration) (Figure 4e,f). Depending on the vibration frequency, the number of black pixels increases up to 4922 at the resonant frequency of 30 Hz, and then decreases down to 0 at 60 Hz, following a negative parabolic relationship. Furthermore, the acceleration dependency of the pattern is quantitatively investigated, as shown in Figure 4f. The number of black pixels is 0 at 3.08 m/s^2^ and gradually increases up to 4871 at 4.60 m/s^2^ along the dot curve. These results indicate that the black patterns on the LCD screen represent vibration information of the target structure, quantitatively enabling decoding of the patterns to vibration frequency and acceleration amplitude.

## 4. Conclusions

In summary, we proposed and demonstrated a PVV sensor system based on the triboelectric effect and a visualization technique. A prototype composed of a PET/Al/LCD screen is fabricated, showing outstanding detection performance as a function of the vibration frequency and acceleration amplitude of the target structure, ranging from 20 to 60 Hz and 3.08 to 4.60 m/s^2^, respectively. In particular, the proposed image processing method is developed to decode the original image pattern to vibration frequency and acceleration values. Therefore, the PVV sensor system can quantitatively calculate the vibration frequency and acceleration from the black patterns on the LCD screen, widely monitoring structural and mechanical vibration of target materials through remote cameras. In addition, this visible light communication system is suitable for nuclear power plants where electromagnetic interference (EMI) by wireless sensor systems is one of the biggest concerns. However, further work is needed to make humidity-resistive PVV sensor to operate in varying environments because the triboelectric charging is sensitive to humidity [33]. Given that the PVV sensor system has advantages of simple conversion-reconversion signal processing and being power and EMI free, it will contribute to the development of self-powered sensor applications, particularly for condition monitoring systems in nuclear power plants [34,35,36].

## Data Availability

Not applicable.

## Supplementary Materials

The following are available online at https://www.mdpi.com/article/10.3390/s21123976/s1, Figure S1: Schematic illustration of fabrication process of the PVV sensor system; (a) Stacking process of Al/PET films. (b) Bonding process of the LCD panel/top part using an adhesive. (c) Al film embedding on the pipe surface. (d) Integration process of top/bottom parts., Figure S2: Figure S2. Power densities of the PVV sensors at the resonant frequencies as a function of load resistance, Video S1: PVV sensors system.
