# Supplementary material for: Patch-Type Vibration Visualization (PVV) Sensor System Based on Triboelectric Effect
Source: Sensors (Basel). 2021 Jun 9;21(12):3976. doi: 10.3390/s21123976 (PMC8227292; doi:10.3390/s21123976)
Supplement: Supplementary file 1 [file sensors-21-03976-s001.zip › sensors-1243513-sup.pdf]

Supplementary Information

Patch-type vibration visualization (PVV) sensor system based on triboelectric effect

Sun Jin Kim[†,1,*], Myeong-Lok Seol[†,2], Byun-Young Chung[1], Dae-Sic Jang[1], Jonghwan Kim[1] and Young Chul Choi[1]

[1] *Smart Structural Safety and Prognosis Research Division, Korea Atomic Energy Research Institute, 111 Daedeok-daero 989Beon-gil, Yuseong-gu, Daejeon 34057, Republic of Korea*

[2] *Center for Nanotechnology and Universities Space Research Association, NASA Ames Research Center, Moffett Field, California 94035, United States*

[†] *These authors contributed equally to this work*

[*] *Correspondence: yesiamtoo@kaeri.re.kr*

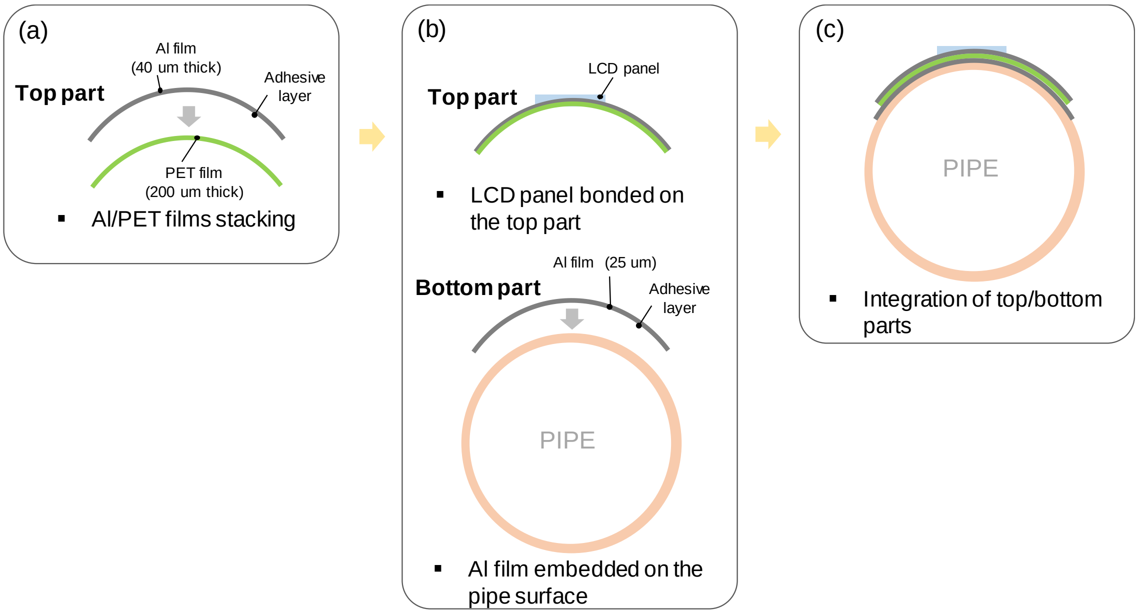

Figure S1. Schematic illustration of fabrication process of the PVV sensor system; (a) Stacking process of Al/PET films. (b) Bonding process of the LCD panel/top part using an adhesive. (c) Al film embedding on the pipe surface. (d) Integration process of top/bottom parts.

The integration process begins with stacking Al (40 µm thick)/PET (100 µm thick) films. After that, LCD panel is bonded onto the Al/PET film (top part). Meanwhile, a Al film is embedded onto the pipe surface (bottom part). Finally, the top and bottom parts are integrated.

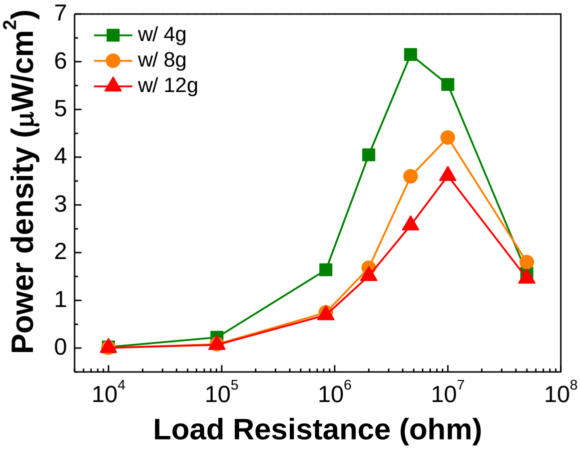

Figure S2. Power densities of the PVV sensors at the resonant frequencies as a function of load resistance.
